# Supplementary material for: Toxoplasma gondii seropositivity among patients with sickle cell disease: Prevalence and association with blood transfusion history
Source: PLoS One. 2026 Apr 3;21(4):e0346155. doi: 10.1371/journal.pone.0346155 (PMC13048495; doi:10.1371/journal.pone.0346155)
Supplement: S1 Questionnaire — (DOCX) [file pone.0346155.s001.docx]

# **QUESTIONNAIRE**

**SECTION 1: SOCIODEMOGRAPHIC CHARACTERISTICS**

**Index #___________ Initials __________**

1. Age _________

2. Gender? Male___ Female____

3. Education status None____ Primary school____ JHS ____ SHS________ Tertiary_______

4. Marital status? Single_____ Married_____ Divorced____ Widowed____

5. Do you have NHIS or private health insurance? Yes__No____ Not sure____

6. Occupation Status. Unemployed____ Student_____ farmer_____ trader______ fisher folk____ civil/public servant________ artisan_____

7. Your/parents Approximate monthly income (cedis) < 500____ 501 - 1,000_____ 1,001-2,000 2,001 - 3,000____ >3,000

8. Source of water supply for drinking? Pipe-borne____ sachet water ___Stream______ River_____

9. Where do you reside? __________________

10. Type of locality Urban________ Rural______

**SECTION 2: THE PREVALENCE OF BLOOD TRANSFUSIONS AMONG THE SICKLE CELL DISEASE PATIENTS**

1. What is your sickle cell status? HbSS___ HbSC__HbSβ⁰______HbSβ⁺_____
2. Are you on Hydroxyurea? Yes No_____ How long_____

2. Have you ever received a blood transfusion? Yes__ No______

If yes:

3. What specific reasons were given for your blood transfusions? Acute pain crisis _____ chronic anemia_________ Other complications_______

Can't recall or not informed_______________

4. In the past 12 months how many times on the average do you receive blood transfusion every year? _______________

5. How many times do you receive blood transfusion every month? ____

**SECTION 3: AN ASSESSMENT OF THE RISK OF ORAL TRANSMISSION OF *T. GONDII* INFECTION AMONG SICKLE CELL DISEASE PATIENTS**

1. Do you own or ever owned a cat Yes_____ No____

If yes:

1.1. Cat living arrangements. Indoors______ Outdoors_____

1.2. Cat defecation. Inside_______ Outside______

1.3. How do you clean cat feces? Sweep____ Pick up with hand while wearing rubber gloves__________ Wash with water_________

3. Stray cats in your compound. Yes_______ No_______

4. Frequency of visiting places with cats. Rarely____ Occasionally_______ Frequently____________

5. Do you ever consume poorly cooked meat, including pork, beef, chicken, or fish? Yes____ No____ Do you eat sausage Yes____ No____

6. Which animal(s) do you consume? – Goat___ Sheep____ Pigs____ Cat____ Chicken_______- Other (………………)

7. How often do you practice hand hygiene, especially before eating?

- Rarely_____ never____ Occasionally_______

- Frequently________

8. Where you live is; walled_____ fenced____ or open____

9. Is the compound of your residence cemented, tiled or covered with pavement blocks? yes_____ no____
