# Supplementary material for: Toxoplasma gondii seropositivity among patients with sickle cell disease: Prevalence and association with blood transfusion history
Source: PLoS One. 2026 Apr 3;21(4):e0346155. doi: 10.1371/journal.pone.0346155 (PMC13048495; doi:10.1371/journal.pone.0346155)
Supplement: Attachment — Submitted filename: RESONSE TO REVIEWERS and editors PLOS ONE.docx [file pone.0346155.s004.docx]

Response to Reviewers

We the authors are very grateful to the reviewers of our manuscript. We acknowledge the effort and time put into this review to make our study better,

Find below comments to all the issues raised.

Reviewer #1: 1. Methods should clearly state which test (ELISA vs RDT) defines seropositivity for the association analyses.

**RESPONSE: we considered both the seropositivity for RDT AND ELISA using ELISA as the reference standard. See Page 14 line # 299-300**

If ELISA is the reference standard, all inferential analyses (logistic regression) should use ELISA-defined seropositivity.

**RESPONSE: This was done. Logistic regression table 6 is stated clearly as logistic regression for the odds of ELISA positive for *t. gondii* among patients with SCD. See page 30 line 513-514**

Currently Tables and text sometimes mix RDT and ELISA and this is confusing.

**Response: yes table 4 and 5 has reports for both RDT and ELISA for chi-square analysis because we want to transparently display the association for both tests, with ELISA as the reference.**

2. The authors imply that past transfusions lead to seropositivity. This is not provable in a cross-sectional design because timing of infection relative to transfusion is unknown. Reword conclusions and discuss that transfusion is an exposure that is consistent with but not proven to be the transmission route.

**RESPONSE: thank you very much. This has been stated in method (study design, page 7, line # 144-147) the discussion (PAGE 37, line # 665-668) and conclusions (page 38, line # 685-687)**

3. Define precisely: “ever had blood transfusion” vs “transfusion in past 12 months” (both appear in the manuscript). Use one primary exposure and justify. If both are used, show separate models.

**RESPONSE: this has been done; transfusion in the past 12 months has been removed. After consideration authors believe that the primary exposure of interest in the “ever had blood transfusion” and “transfusion in past 12 months” is redundant**

4. For participants with transfusion in the past 12 months, consider sensitivity analysis excluding individuals whose seropositivity might have preceded transfusion (if dates available) or at least stratify by time since last transfusion.

**RESPONSE: this is no more necessary since we have removed the “transfusion in past 12 months”**

5. For every table and subgroup report the exact n used. If any missingness exists for covariates or outcomes, report number and pattern; explain handling (complete-case only, imputation).

**RESPONSE: there were no missing data in this study. Care was taken during collection of data for all 156 patients. However, where appropriate, the exact n used in the table have been reported**

6. Table 4 & 5: For subgroup comparisons (age categories, genotype, etc.) provide risk differences and 95% CIs or prevalence ratios rather than p-values alone. This gives clinical meaning to findings.

**RESPONSE: this has been done prevalence ratios and 95% CIs have been added as advised**

7. Use Fisher’s exact test when expected cell counts <5. For example, groups with very small n (age ≥60) must use Fisher’s exact or combine categories.

**RESPONSE: combined categories have been done thus chi-square is more appropriate. Thank you**

9. Provide a priori rationale for covariates included in the adjusted model. Avoid data-driven “stepwise” selection unless justified; if used, report selection procedure and results.

**RESPONSE: this has been done. see data analysis section of methodology. Page 14 line # 308-315**

10. For each fitted logistic model, report:
• Number of events and non-events (EPV — events per variable) to ensure model is not overfitted. With 83 ELISA positives, be cautious about the number of covariates in adjusted model (rule of thumb: >10 events per parameter).

**RESPONSE: categories in the variables in the logistic regression model have been revised to reduced predictors to 5. Thus, with events as 83 the EPV is 16.6 which is acceptable. See page 14 line # 316-320 (page 15)**

• VIFS FOR MULTICOLLINEARITY.
• Hosmer–Lemeshow result
• ROC AUC with 95% CI.

RESPONSE: the above were all satisfactory.

- **VIFs** confirm no collinearity issues.
- **Hosmer–Lemeshow** shows the model fits the data well.
- **ROC AUC** indicates strong predictive ability.

11. Investigate interaction between age and blood transfusion (biologically plausible). If interaction is present, present stratified ORs.

**RESPONSE: thank you very much for this suggestion. In figure 1 the distribution and association were shown with an insignificant p value of 0.28 however, the authors believe this association is the not the focus of this manuscript, thus there was no need to investigate futher.**

13. Provide the full 2×2 table in the main text or supplement (you have provided TP/TN/FP/FN but ensure clarity).

**RESPONSE: 2 by 2 table has been added, see page 22**

State whether ELISA is true gold standard or imperfect reference and discuss implications.

**RESPONSE: ELISA is an imperfect reference. Implication discussed in the discussion section see page 33 line # 570-573**

14. Report exact binomial (Clopper–Pearson) CIs for sensitivity/specificity and predictive values; indicate the method used to compute CIs.

**RESPONSE: this has been done. see page 22 line # 438-440**

15. Kappa = 0.634 is “substantial” agreement; state the scale used for interpretation (e.g., Landis & Koch).

**RESPONSE: this has been done. see page 22 line # 438**

16. Discuss that PPV/NPV depend on prevalence and provide context (e.g., expected values in general population).

**RESPONSE: this has been done. page 34 see line # 585-590**

Reviewer #2: Dear Editor,
In a cross-sectional study conducted with 156 patients suffering from sickle cell disease (SCD) at Ho Teaching Hospital in the Volta Region of Ghana, the authors observed that the seroprevalence of Toxoplasma gondii, detected by ELISA, was significantly higher at 53.0% compared to 38.5% detected by rapid diagnostic tests (RDT). Furthermore, a history of blood transfusion was associated with an increased likelihood of T. gondii seropositivity among SCD patients, suggesting that transfusions may represent a potential route for transmission within this population.
Several questions arise from this study:
- Were consecutive SCD (sickle cell disease) patients enrolled, or is there potential for sampling bias?

R**RESPONSE: yes, consecutive sampling technique was used in this study THIS HAS BEEN stated in the methodology see line # 185-192, PAGE 9**

- Were any exclusion criteria applied that might influence seroprevalence?

**RESPONSE: Exclusion criteria have been stated see line # 199-199, PAGE 9**

- How were discordant IgG and IgM results managed?

**RESPONSE: discordant IgG and IgM was not a problem in this study since seropositivity was defined as positive for either IgM, IgG or IgM and IgG combination for both RDT (see line # 264-265, page 12) and ELISA. (line # 285-286, page 13)**

- A positive IgG result indicates past exposure to the infection but does not confirm an active infection. To distinguish the timing of infection, IgG avidity testing should be performed.

Response: yes. This basic fact was acknowledged in the discussion section **see line # 640-643, Page 36. Unfortunately, IgG avidity testing was not done in this study aside the financial constraints, the focus was on overall exposure or evidence of infection, rather than on timing of the infection. See line # 283-285, Page 13**

- Why didn't you use molecular tests to detect active infections?

**RESPONSE: again, budgetary constraint was a factor**

- How was transfusion history defined in this study?

**RESPONSE: this was defined as having ever received one or more transfusions of any type, with responses categorized as “yes” or “no.” see line # 222-224, page 10**

- Were potential confounders included in the analysis model?

**RESPONSE: yes. these included age, cat ownership, consumption of undercooked or raw meat, and educational status. see line # 308-315, page 14**

- What is the prevalence of Toxoplasma gondii in the general population?

**RESPONSE: in the volta region where this study took place no prevalence has been reported in literature (see page 6 124-126). However, in Ghana prevalence have been reported as high 70% in pregnant women (see page 6, line 119-121)**

- How is transfusion interpreted as a transmission route, given the cross-sectional design?

**Response: yes, this study is a cross-sectional design which can only determine association but not causality. So, this study cannot categorically say that the patients who were seropositive got the infection from blood transfusion. This has been stated in method (study design, page 7, line # 144-147) the discussion (PAGE 37, line # 665-668) and conclusions (page 38, line # 685-687)**

- How is the discrepancy between ELISA and RDT prevalence reconciled?

**RESPONSE: ELISA was the reference standard. RDT performance was compared with ELISA. Results of RDT and ELISA were presented side by side in table 4 and 5 but inferential statistics such as logistic regression analysis relied solely on ELISA. The observed discrepancy was adequately discussed see page 33 line # 568-590 (page 34)**

- Were the results communicated to the clinic or to the patients, and was follow-up offered for those who tested positive?

**Response: All participants were educated on toxoplasmosis. Results were communicated to participants and those who tested positive were further counseled and follow up initiated by the attending physician see line # 328-333, page 15**

Reviewer #3: Dear editor
Thank you for the opportunity to review the manuscript titled “seroprevalence of Toxoplasma gondii among sickle cell disease (SCD) patients in Ghana”. I would like to appreciate the authors' effort to undertake this valuable task”. The study investigates the seroprevalence of Toxoplasma gondii among sickle cell disease (SCD) patients in Ghana, with emphasis on the potential association between blood transfusion history and seropositivity. The topic is clinically relevant and fills a local research gap, especially since transfusion-transmitted T. gondii infections are poorly characterized in sub-Saharan Africa.

**Response: Thank you very much**

However, despite its importance, the study has several methodological and presentation flaws that undermine the robustness and reproducibility of the findings. Major revisions are required before the paper can be considered for publication in a journal such as PLOS ONE.
Major Comments
Study Design and Objectives
The cross-sectional design is appropriate for prevalence estimation but cannot establish causality or temporal relationships between transfusion and infection. The authors acknowledge this in the discussion, but they should emphasize it clearly in the Abstract and Methodology.

**Response: this has been stated in abstract (page 3 line # 69-70) , method (study design, page 7, line # 144-147) the discussion (PAGE 37, line # 665-668) and conclusions (page 38, line # 685-687)**

The objective (“to find out the prevalence of T. gondii infection and the association of blood transfusion among patients with SCD”) is acceptable but could be phrased more precisely as:
“To determine the prevalence of anti-Toxoplasma gondii antibodies among SCD patients and to assess the association between seropositivity and history of blood transfusion.”

**Response: this has been done see line # 129-130 page 6**

Sample Size and Sampling Procedure
The sample size calculation using Cochran’s formula is correctly presented, but the population (N=300) was taken from clinic attendance, not total SCD population in the region—this introduces selection bias. The manuscript lacks details on sampling technique (e.g., consecutive sampling, random selection). Specify how participants were recruited to ensure representativeness.

**Response: yes, consecutive sampling technique was used in this study. This has been stated in the methodology see line # 185-192, PAGE 9**

Diagnostic Testing
The use of RDT and ELISA is appropriate, but the RDT performance characteristics should have been validated with a local control group or WHO-standard panels to confirm accuracy.

**Response: Unfortunately, this was not done due to budgetary constraints. However, we used ELISA a widely accepted superior test, as reference standard to complement and assess the performance of RDT findings. We the authors believe using ELISA reference standard can suffice as a pragmatic internal reference validation. The performance of RDT has been stated in the methodology section (page 14 line # 299-300) results section, table 3, page 21, line # 431-440 (page 22) and discussion section see page 33, line # 568-593 (page 34)**

Details on kit lot numbers, manufacturer references, and quality control measures are missing. For reproducibility, these must be stated.

**RESPONSE: THIS HAS BEEN stated. See page 11, line # 240-248**

Defining “seropositive” as IgG or IgM or both is acceptable, but distinguishing recent (IgM) from past (IgG) infections would have improved epidemiological interpretation.

Response: Yes, this accurate. However, while IgM and IgG suggest recent and past infections respectively this is not always the case as IgM can persist for a long time and Ig G might not be precise in the timing of the past infection. This calls for another test such as IgG avidity test which could accurately establish past and current infection. Unfortunately, this was not done in this study due to budgetary constraints How ever **the focus of this study was on overall exposure or evidence of infection, rather than on timing. See page 13 line # 283-285**

Data Analysis
The analysis using SPSS v25 is fine, but the variable coding and handling of missing data are not described.

**RESPONSE: this has been stated. See page 13 line # 293-298 (Page 14)**

The logistic regression model should include confidence intervals for all variables, not only blood transfusion.

Response: All variable in table 6 have their confidence interval thank you page 30 line 513-524

Also, justification for including variables in the multivariate model should be given (based on univariate p<0.2, for example).

Response: the main criteria used here is based on a priori theoretical and clinical rationale, supported by prior literature. However aside cat ownership and consumption of undercooked or raw meat the variables used had significant association in chi-square analysis. **This has been stated. see data analysis section of methodology. Page 14 line # 308-315**

The adjusted OR (3.14) is significant but could be confounded by age and exposure factors not properly adjusted for. Sensitivity analyses would strengthen the claim.

**Response: Yes, Sensitivity analyses such as stratification or alternate model specifications would have strengthen the claim, but were limited by sample size. We have added this as a limitation in the Discussion. SEE LINE 669-671, PAGE 37**

Results Interpretation
The results are clearly tabulated, but some tables are overcrowded (e.g., Table 4 and 5) — consider splitting into demographic vs. clinical characteristics.

Response: Thank you very much. However, we the authors believe table 4 and 5 are ok especially with the revision made and table now put in lands scape orientation.

The conclusion that blood transfusion may transmit T. gondii is overstated. Seropositivity does not confirm transmission via transfusion; longitudinal or donor-recipient studies are needed.
Discussion should better distinguish between association and causation, and avoid speculative statements without molecular evidence.

**Response: this has been stated. see line 665-668 page 37 and line 685-689, page 38**

Language and Style
Numerous grammatical, typographical, and stylistic errors (e.g., “varried,” “haemoglobinopathy,” “lectrophoresis”) need correction by a professional English editor.
The tone should be more scientific and concise; many sentences in the Introduction and Discussion are repetitive or verbose.

**Response: This has been done thank you very much**

References are inconsistently formatted — ensure compliance with PLOS ONE reference style (DOI, complete author list, year, journal, volume, pages).

**Response: this has been done thank you very much**

Ethical and Data Availability Statements
Data availability statement (“data cannot be shared publicly because it was generated from patients attending the Ho Teaching Hospital”) does not meet PLOS ONE’s open data policy. A controlled-access mechanism (e.g., through institutional data office) should be mentioned.

**Response: Data has been provided thank you**

Minor Comments
Abstract:
Include numeric results for odds ratios and p-values.

**Response: This has been done thank you**
Avoid abbreviations like “RDT” without prior definition.

**Response: This has been done .Thank you**
Figures:
Figures 1–3 are informative but should include error bars and legends. The quality should be improved for publication.

Response: This has been done
Tables:
Add column totals and indicate statistical tests (e.g., χ², Fisher’s exact) in captions.

Response: Adding column total will make the tables more crowded especially since it has been revised to include prevalence ratio column

χ² has been indicated for table 4 and 5.

For clarity, present p-values to three decimal places consistently.

Response: This has been done
Introduction:
Reduce literature overload; several global prevalence citations could be summarized more succinctly.

Response: This has been done thank you
Add rationale for selecting SCD patients beyond frequent transfusion — e.g., immunocompromised state.

Response: Thank you very much for this suggestion. while we the authors agree with the immunocompromised state among SCD patients, the sole rational for selecting SCD patients in this study is their relationship with blood transfusion.

Discussion:
Avoid restating the results; instead, focus on comparative analysis with prior studies.

Response: This has been done thank you

Include a short section on public health implications, e.g., the need for donor screening for T. gondii in Ghana.

**Response: This has been done thank you page 37 line # 652-657**

Conclusion:
Should emphasize limitations and recommend future longitudinal or molecular studies.

**Response: This has been done thank you**
