# Supplementary material for: Toxoplasma gondii seropositivity among patients with sickle cell disease: Prevalence and association with blood transfusion history
Source: PLoS One. 2026 Apr 3;21(4):e0346155. doi: 10.1371/journal.pone.0346155 (PMC13048495; doi:10.1371/journal.pone.0346155)
Supplement: Attachment — Submitted filename: Response to Reviewers2.docx [file pone.0346155.s005.docx]

RESPONSE TO REVIEWERS

We sincerely thank the reviewers for their painstaking effort in improving our manuscript through valuable comments and constructive suggestions. We have carefully considered each point raised and provide detailed responses below. In revising the manuscript, we have incorporated the reviewers’ insights to enhance clarity, strengthen the methodology, and improve the overall quality of the paper.

Below, we present our responses to the specific issues raised:

Reviewer #1:

 • The primary outcome should be clearly defined as ELISA-defined seropositivity.

Response: this the main focus of the study. It has now been clearly stated in the manuscript. See pages # 12 line # 289-290 **“For this study, seropositivity was operationally defined using ELISA results, and this served as the primary outcome of interest**”.

Analyses using RDT results should be limited to reporting diagnostic performance and should not be used as the outcome in regression models to avoid misinterpretation due to misclassification.

Response: ELISA was used as the primary outcome of regression model and not RDT. See data analysis pages # 13 line # 311-312 “**Multiple logistic regression was performed to estimate the association between blood transfusion and the odds of T. gondii ELISA seropositivity”** and result pages # 27 line # 498-499 “**Multiple logistic regression analysis was further carried out on variables with significant association with ELISA resuts to find out the odds of positive ELISA results among the SCD** ptients”

State the seropositivity definition in the Methods section and reference the ELISA cutoff per manufacturer; reserve RDT results for supplementary sensitivity analyses.

Response: this has been stated See pages # 12 line # 284-285 “**To determine the critical cut-off, the average absorbance of the negative controls was summed with 0.15; page # 12 line # 289-290 “ELISA positive is defined as positive for either IgM, IgG or IgM and IgG combination.”**

• Choose a single primary exposure (e.g., ever transfused or transfusion in the past 12 months) with a priori justification.

Response: This has been done. “Transfused in the past 12 month” was considered to be redundant and has been appropriately deleted See pages # 9 line # 226-228 “**Information on the lifetime history of blood transfusion was obtained. This was defined as having ever received one or more transfusions of any type, with responses categorized as “Yes” or “No.”.**

• Present full diagnostic metrics for the adjusted model: variance inflation factors (VIFs) for multicollinearity, Hosmer-Lemeshow goodness-of-fit, area under the curve (AUC) with 95% confidence interval, and calibration metrics (e.g., Brier score or calibration plot).

Response: We thank the reviewer for the valuable suggestion to present full diagnostic metrics for the adjusted prediction model. In the revised manuscript, we have now included comprehensive model validation results and **added all requested diagnostics. See page # 28 line # 512-522**

• Interaction between age and transfusion was considered; the authors decided not to pursue it due to a non-significant p-value in the figure. Pre-specify a clinically plausible interaction (e.g., age × transfusion) and test it in the full model. Report stratum-specific odds ratios if interaction is present. If not, state that the interaction was not supported by the data and discuss clinical relevance.

Response: We appreciate the reviewer’s comment. In the revised manuscript, we have clarified that a clinically plausible interaction between age and transfusion was pre‑specified and tested in the full multivariate model. The interaction term was statistically significant (p = 0.02), and stratum‑specific odds ratios were therefore reported see method/analysis section page # 13, line # 327-330; result section page # 29 line # 536-541; discussion page 32 line # 626-632	52

• Ensure tables (Table 1 and Table 2) include column totals where helpful, with explicit test names in captions.

Response: columns total has been added to table 1 and 2. These tables summarize the background profile of the SCD patients (age, sex, education, genotype, transfusion history, etc.) but they do **not** present diagnostic test results (RDT or ELISA outcomes)

• Throughout the text, use "multiple logistic regression" instead of "multivariate logistic regression."

Response: this has been done, thank you very much

Reviewer #4: Title: Toxoplasma gondii seropositivity among patients with sickle cell disease: Prevalence and Association with Blood Transfusion History

Comment to authors

General comment
Authors dedicate the comments of all reviewers but some are still need to get more explanation. Title is more prefer to the epidemiological study but the methods are combined with diagnostic accuracy study. The reviewers also give the comment for confusing the usage both RTD and ELISA for assessing the seropositive [already known that the ELISA is more accurate for detection than the RTD – to align with the title, just suggest 1- removing the result of accuracy finding,

2- all analysis with reference diagnosis (ELISA) by setting the operational definition of seropositive.]

Response: We appreciate the reviewer’s comment regarding the alignment of the title with the study scope. We have retained the original title, *“Toxoplasma gondii seropositivity among patients with sickle cell disease: Prevalence and Association with Blood Transfusion History,”* because the primary focus of the study is epidemiological — estimating prevalence and examining associations with transfusion history.

We also included RDT results to evaluate diagnostic accuracy against ELISA, given the widespread use of RDTs in resource‑limited settings. This secondary analysis broadens the relevance of the study by providing practical insights into diagnostic performance, while not altering the epidemiological focus signaled in the title. The Methods and Results are aligned to make this distinction explicit, ensuring clarity for readers.

Clearly stated in the Methods, ELISA‑defined seropositivity was the operational definition and the primary outcome for all prevalence and association analyses.

*“****For this study, seropositivity was operationally defined using ELISA results, and this served as the primary outcome of interest..*** (Methods, ELISA procedure See pages # 12 line # 289-290)

*“****The diagnostic performance of the rapid diagnostic test (RDT) was evaluated against ELISA as the reference standard* Methods”, (Data Analysis page # 12 line # 302-303*… Multiple logistic regression was performed to estimate the association between blood transfusion and the odds of T. gondii* ELISA seropositivity.” ( Methods, Data Analysis page # 13 line 313-314)**

.

Specific comment
Comment 1: Abstract, p value should be consistent in decimal (suggested three decimal)

RESPONE: This has been done thank you very much

Comment 2: Abstract, the sentence of “This cross-sectional design allowed estimation of prevalence and associations, but not causality or temporality. More robust longitudinal studies are needed to help elucidate the true contribution of blood transfusion transmission of T. gondii” should be removed to limitation in discussion.

Response: We appreciate the reviewer’s comment regarding the placement of the limitation sentence. While we acknowledge the suggestion, this statement is already in the limitation section of discussion (see the highlighted texts on page # 34 line # 663-678).

However, we have chosen to retain it in the Abstract in agreement with a previous reviewer’s recommendation, but revised it into a concise sentence. We believe that acknowledging this limitation at the Abstract stage not only provides readers with immediate clarity on the scope of the study but also constructively emphasizes the need for future longitudinal or interventional studies to elucidate the role of blood transfusion.

Comment 3: Abstract, the recommendations based on significant associations should be added in conclusion.

Response: We thank the reviewer for this valuable suggestion. In line with the significant associations observed in our study, we have revised the Abstract conclusion to include clear recommendations.

Specifically, we now highlight that the strong association between *T. gondii* seropositivity and blood transfusion underscores the need to strengthen transfusion safety protocols and consider screening strategies for *T. gondii* among high‑risk populations such as patients with sickle cell disease.

Comment 4: Study Design, “study design was employed to estimate the prevalence of T. gondii infection and to examine associations with blood transfusion history. While suitable for prevalence estimation and association testing, this design cannot establish causal or temporal relationships.” Is nearly the same with Line No. 105-107 and these sentences should be removed.

Response: We thank the reviewer for this observation. We agree that the sentences are redundant and they have been deleted. Thank you very much.

Comment 5: Study Area and Site, the subheading should be “Study Area”.

Response: Thank you very much, this has been done

Comment 6: Sample size determination, please recheck the sample size (would be 169?).

Response: We thank the reviewer for pointing out this issue. On re‑checking our calculation, we realized that the population size was incorrectly stated as 300 in the initial draft. The correct number of registered sickle cell disease patients at the clinic is 210. Using Cochran’s formula with $p=0.5$, $Z=1.96$, and allowable error of 0.05, the initial sample size was 385. Applying the finite population correction for $N=210$gave a minimum sample size of 136. To increase the power of the study, we recruited 156 patients, which exceeds the minimum required. We have corrected this section in the Methods to reflect the accurate calculation and population size.

Comment 7: Sample size determination, can you explain the usage of n = n/ 1+(n/N) instead of n = n/ 1+(n-1/N) as Cochran’s modified formula for finite populations?

Response: thank you for raising this important point. In our initial draft, we presented the finite population correction formula in a simplified form as $n=\frac{n_{0}}{1+\frac{n_{0}}{N}}$. We acknowledge that the precise Cochran’s modified formula is $n=\frac{n_{0}}{1+\frac{n_{0}-1}{N}}$. The difference between these two versions is negligible when $n_{0}$is large, as in our case ($n_{0}=385$), but for rigor and clarity we have corrected the Methods section to reflect the exact formula. $n=\frac{n_{0}}{1+\frac{n_{0}-1}{N}}$.

Using the corrected formula with $N=210$registered sickle cell disease patients, the minimum sample size is 136. We ultimately recruited 156 patients, which exceeds the minimum required and ensures adequate study power. We have revised the Methods section accordingly to present the correct formula and calculation step‑by‑step.

Comment 8: Sampling and Recruitment Procedure, please revise as “Given the relatively small clinic population of 300 registered patients, a consecutive sampling was employed, where all eligible SCD patients attending the clinic during the study period were recruited sequentially until the required sample size was achieved” in Line No. 162-165.

Response: Thank you very much for this suggestion. This has been revised as instructed

Comment 9: Figure 1 and 2, please specify visualization to clear for describing the type of variables. Error bars are reliable for numerical data and should not be add in description of categorical or nominal data. (numerical data - bar for mean and error bar for its SD or SE, categorical or nominal data - bar for number or percent and error bar for 95% CI of percent).

Response: Thank you for highlighting the need to align error bars with the type of variable. In the revised manuscript, Figures 1 and 2 have been updated to ensure clarity and consistency with statistical conventions. Specifically, both figures present categorical variables (blood transfusion distribution and risk factors). Accordingly, the bar charts now display percentages with error bars representing the 95% confidence intervals of those percentages, rather than standard errors of the mean.
