# Supplementary material for: Toxoplasma gondii seropositivity among patients with sickle cell disease: Prevalence and association with blood transfusion history
Source: PLoS One. 2026 Apr 3;21(4):e0346155. doi: 10.1371/journal.pone.0346155 (PMC13048495; doi:10.1371/journal.pone.0346155)
Supplement: Attachment — Submitted filename: response3 to Reviewer 1.docx [file pone.0346155.s006.docx]

**RESPONSE TO REVIEWERS**

**We the author once again want to thank the editor and reviewers for the guidance so far. Thank you very much for the comments. kindly find below response to the issues raised.**

**Reviewer #1: The suggested revisions to Table 1 and Table 2 are intended to improve the clarity and interpretability of the data distributions. Specifically:

A total column should be added to summarize the overall frequency or percentage across all categories for each explanatory variable.
Two separate columns should be included to show the distribution of each explanatory variable stratified by the levels of the outcome (response) variable.

This structure will allow readers to clearly see both:
(a) the conditional distribution of each predictor across outcome strata, and
(b) the marginal (overall) distribution of each predictor in the entire sample.

Furthermore, it is strongly recommended that:

Column totals be included where they meaningfully contribute to data interpretation.
The exact names of the statistical tests used (e.g., chi-square test, Fisher's exact test, trend test) be explicitly stated in the table captions to ensure transparency and reproducibility.**

**Response to Reviewer #1:**

We appreciate the reviewer’s thoughtful recommendations to restructure Tables 1 and 2 by adding total columns and stratified columns for outcome variables. While we fully agree with the importance of clarity and interpretability, we respectfully propose to retain Tables 1 and 2 in their current format as descriptive summaries of the overall (marginal) distributions of sociodemographic and clinical characteristics. This approach allows these tables to serve as baseline descriptive data for the study population. However, table 4 and 5 provided some of the information

To address the concern, we have:

1. **Added total columns** to Tables 4 and 5 to summarize the overall frequency and percentage across categories, thereby improving clarity.
2. **Directed readers to Tables 4 and 5**, which already present the stratified (conditional) distributions by *T. gondii* RDT and ELISA seropositivity, along with prevalence ratios (PR), confidence intervals (CI), and p‑values.
3. **Revised captions and narrative text** to explicitly state that Tables 1 and 2 show marginal distributions, while Tables 4 and 5 show conditional distributions.
4. **Specified the exact statistical tests used** (Pearson’s chi‑square or Fisher’s exact test where expected cell counts were <5) in the captions of Tables 4 and 5, ensuring transparency and reproducibility.

We believe this structure maintains clarity, avoids redundancy, and ensures that both marginal and conditional distributions are clearly presented in the manuscript.
